# Serum klotho: a potential predictor of cerebrovascular disease in hemodialysis patients

**DOI:** 10.1186/s12882-019-1232-2

**Published:** 2019-02-21

**Authors:** Honglan Wei, Hua Li, Xiaohong Song, Xingguo Du, Yuan Cai, Chengxu Li, Liping Dong, Junwu Dong

**Affiliations:** Department of Nephrology, Puai Hospital, Tongji Medical College, Huazhong University of Science and Technology, Wuhan, Hubei 430033 People’s Republic of China

**Keywords:** Hemodialysis, Klotho, Cerebrovascular disease, Cognitive impairment

## Abstract

**Background:**

Hemodialysis patients suffer from a serious threat of cerebrovascular disease. Klotho, as an aging-suppressor gene, contributes to protect on vascular calcification and oxidative stress, which are the risk factors of cerebrovascular disease. The purpose of the present study is to determine the relationship between serum klotho and cerebrovascular disease in patients receiving hemodialysis.

**Methods:**

Serum klotho levels of hemodialysis patients were measured by ELISA. Cerebrovascular diseases were diagnosed by CT or MRI scans. The cognitive function of hemodialysis patients with cerebrovascular disease were evaluated with a neuropsychological battery assessing domains of global cognition verbal memory, spatial memory, executive function and verbal fluency.

**Results:**

Eighty-eight patients were included, 57 ± 14 years, 63.64% male, 52.27% older than 60 years. Twenty-eight participants had cerebrovascular disease (23 cases had cerebral infarction, 5 cases had cerebral hemorrhage). The average level of serum klotho of all participants was 119.10 ± 47.29 pg/ml. The serum klotho level was significantly associated with cerebrovascular disease in hemodialysis patients (HR(95%CI) = 0.975(0.960–0.990), *p* = 0.001). The optimal cut-off value of serum klotho for predicting cerebrovascular disease in hemodialysis patients was 137.22 pg/ml, with a specificity of 96.4% and a sensitivity of 46.7%. But serum klotho was not an independent risk factor of cognitive impairment for hemodialysis patients with cerebrovascular disease (HR((95%CI) = 1.002(0.986–1.018), *p* = 0.776) or with cerebral infarction (HR(95%CI) = 1.005(0.987–1.023), *p* = 0.576).

**Conclusions:**

The serum klotho level is a potential predictor of cerebrovascular disease in hemodialysis patients, but it is not an independent risk factor of cognitive impairment for hemodialysis patients with cerebrovascular disease.

## Background

Over the past decade, chronic kidney disease (CKD), defined as a decreased glomerular filtration rate or increased albuminuria is recognized as a worldwide important public health problem, which is highly prevalent in developing countries. China is the largest developing country. In Chinese adults, the prevalence of CKD is 10.8% [1]. It means that there are about 119.5 million Chinese aged 18 years or older have CKD [1]. With the prevalence of CKD, end-stage renal disease (ESRD) has become an emerging worldwide health burden because of its many complications, high costs and mortality risk. Renal replacement therapy, containing hemodialysis, peritoneal dialysis and kidney transplant, is the main treatment for end-stage renal disease currently. There are about 2.2 million end-stage renal disease patients receiving renal replacement therapy, and hemodialysis accounts for 89%. In China, 454,000 patients are receiving hemodialysis in 2016, where the number is 276, 008 in 2011.

Hemodialysis is associated with raised risk of cardiovascular disease (CVD), infection, cerebrovascular disease and other complications. The risk of cerebrovascular disease in patients receiving hemodialysis is six to ten times higher than in the general population [2]. Cerebrovascular disease is the third leading cause of death for patients on dialysis [3]. In excess of 35% dialysis patients with cerebrovascular disease experience short-term mortality [4]. Except that, disability and cognitive impairment are very common in survivors [5, 6]. Cerebrovascular disease is prevalent in hemodialyis patients partly related to the traditional risk factors, such as age, hypertension, diabetes mellitus and dyslipidemia. The uremic and dialysis process itself also the independent risk factors for cerebrovascular disease [7, 8].

Calcium-phosphate imbalance plays a crucial role in vascular calcification, which is the risk factor of cerebrovascular disease. Klotho protein, which is predominantly expressed in the kidney and brain, has a unique role in renal calcium and phosphorus metabolism [9–11]. Furthermore, klotho contributes to protect on vascular calcification and oxidative stress [12, 13]. Therefore, decreased klotho protein could be a novel non-traditional risk factor for cerebrovascular disease in hemodialysis patients.

Based on the strong vascular-protective effects of klotho, the current study aimed to confirm the relationship between serum klotho and cerebrovascular disease in patients receiving hemodialysis. It is hypothesized that decreased serum klotho protein is the novel risk factor of cerebrovascular disease in hemodialysis patients.

## Methods

### Participants

From November 2014 to March 2015, we recruited participants from the Dialysis Center in Puai hospital of Tongji medical college, Huazhong University. Eligible participants were 18 years or older, receiving maintenance hemodialysis for at least 3 months, and had undergone head CT or MRI examination in the past 1 year which excluded the history of stroke or silent brain infarction (SBI). Cerebral hemorrhage caused by craniocerebral trauma during the study and patients with stroke history were excluded. Patients were also excluded if they had an active psychiatric disorder, Alzheimer’s disease, or if they had significant visual or hearing impairment. Serum klotho measurement and other laboratory studies were carried out at the beginning of the study. Every participant was followed up for 24 months. If the participants have stroke symptoms, head CT or MRI were scanned immediately. The others without stroke symptoms during the following time, head MRI were carried out in the last month of the study.

### Cerebrovascular disease

Cerebrovascular diseases, including clinical stroke and SBI, were diagnosed by two neurology specialists and two imaging specialists. Clinical stroke were diagnosed through head CT or MRI scans and clinical symptoms which contain sudden onset of unilateral numbness, unilateral weakness, loss of ability to communicate, loss of ability to understand, loss of vision, and loss of half the visual field. SBI was diagnosed through MRI.

### Laboratory studies

The fasting blood samples were collected in the morning and pre-dialysis. After collection, the blood samples were centrifuged immediately, serum and plasma were obtained and stored at-80 °C. Serum klotho was detected by ELISA [14, 15], and antibodies were purchased from Xinfan Bio technology company, Shanghai, China. Automatic biochemical analyzer (Mindray, Shenzhen, China) was used for measuring albumin (ALB), calcium (Ca), phosphate (P), iron, total iron binding capacity (TIBC), total cholesterol (Tch), triglycerides (TG), and high-density lipoprotein cholesterol (HDL-C) concentrations and Low-density lipoprotein cholesterol (LDL-C). Hemoglobin (Hb) was measured by Automatic blood analyzer (Mindray, Shenzhen, China). Serum ferritin (SF) and parathyroid hormone (PTH) was measured by Radioimmunoassay (Beckman Coulter, California, USA).

### Cognitive function

All the patients with cerebrovascular disease were evaluated cognitive function with a neuropsychological battery assessing domains of global cognition verbal memory, spatial memory, executive function and verbal fluency. Global cognition was assessed with the mini-mental state examination (MMSE). Verbal memory was assessed with the Hopkins Verbal Learning Test-Revised (HVLT-R) immediate and delayed recall components [16]. Spatial memory was assessed with the Brief Visuospatial Memory Test-Revised (BVMT-R) [17]. Executive function was assessed with the Trail A test [18]. Verbal fluency was assessed with the Verbal Fluency Test (VFT) [19]. Cognitive impairment was defined as a score at least 1.5 SD below normative values for age in 2 or more cognitive domains [20].

### Statistical analysis

Datas with non-normal distribution were expressed as medians and interquartile ranges (25th and 75th percentiles) or percentages for categorical variables, while others with normal distribution were expressed as mean ± SD. We compared characteristics of patients with cerebrovascular disease to those without using the 2-tailed Student’s *t*-test. Categorical data are presented as numbers and percentages and compared using the Chi-square test.

Cox regression analysis was used to explore the independent risk factors of cerebrovascular disease in hemodialysis patients. A receiver operating characteristic (ROC) curve was performed to examine the prognostic utility of serum klotho and to identify the optimal cut-off value.

The relationship between klotho and cognitive function of patients with cerebrovascular disease was explored using Cox regression models: model 1, crude model; model 2, additionally adjusting for age; model 3, additionally adjusting for diabetes mellitus (DM).

The correlations between the different parameters and klotho levels were evaluated using a correlation test.

All statistical analyses were performed using SPSS (version 20, IBM Corp, Amonk, NY, USA). A *p*-value < 0.05 was considered statistically significant.

## Results

### Participants’ characteristics

A total of 185 patients were screened. The hemodialysis patients with stroke history (*n* = 20) or without head CT or MRI in the past 1 year to certify who had no stroke or silent brain infarction were excluded (*n* = 15). Patients were also excluded if they had an active psychiatric disorder (n = 2), Alzheimer’s disease (*n* = 18), or if they had significant visual or hearing impairment (*n* = 24). Eighteen patients refused to participate in the study. A total of 88 hemodialysis patients were included for the current study, and 56(63.64%) cases were male. The average age of the participating patients was 57 ± 14 years, and 46(52.27%) patients were older than 60 years. 34 (38.64%) cases had diabetes mellitus. Of the participants, 28(31.82%)had cerebrovascular disease (23 had cerebral infarction, 5 had cerebral hemorrhage). The time between the serum klotho measurement and the cerebrovascular disease event was 16.86 ± 8.29 months. In the patients with cerebrovascular disease, 20 (71.43%) cases were male, 15 (53.57%) cases had diabetes mellitus, and 23(82.14%) cases were older than 60 years. The clinical characteristics of participants with cerebrovascular disease were very similar to participants without cerebrovascular disease, except for age and PTH (Table 1). The average level of serum klotho of all participants was 119.10 ± 47.29 pg/ml. As compared to the patients without cerebrovascular disease, serum klotho level was significantly lower in the participants with cerebrovascular disease (91.65 ± 28.19 pg/ml versus 131.90 ± 49.09 pg/ml, *P* = 0.000) (Table 1).

**Table 1:** Characteristics of participants

		With	Without
	Total	cerebrovascular disease	cerebrovascular disease
	(*n* = 88)	(*n* = 28)	(*n* = 60)
Age, years	57 ± 14	66 ± 10	54 ± 14 **
Male, n (%)	56 (63.64%)	20 (71.43%)	36 (60%)
Diabetes history, n (%)	34 (38.64%)	16 (57.14%)	18 (30%)
Hypertension, n (%)	72 (81.82%)	24 (85.71%)	48 (80%)
Tobacco smoking, n (%)	12 (13.64%)	4 (14.29%)	8 (13.33%)
HD vintage (months)	34.55 ± 18.57	40.86 ± 17.09	31.60 ± 18.63
Klotho (pg/ml)	119.10 ± 47.29	91.65 ± 28.19	131.90 ± 49.09**
Hb (g/L)	99.94 ± 15.34	100.93 ± 13.12	99.48 ± 16.36
Ca (mmol/L)	2.19 ± 0.25	2.20 ± 0.23	2.18 ± 0.26
P (mmol/L)	1.90 ± 0.52	1.84 ± 0.56	1.93 ± 0.50
PTH (pg/ml)^a^	460.23 (185.75–538)	343.32 (185.75–478.5)	514.78 (176.50–727.75)*
SF (ng/ml) ^a^	410.59 (112–528)	349.64 (84.5–404.25)	439.51 (121–582)
Serum iron (umol/L) ^a^	10.99 (8.06–13.30)	10.92 (8.24–12.58)	11.02 (7.71–13.52)
TIBC (umol/L)	47.10 ± 9.16	46.95 ± 9.65	47.18 ± 9.00
Tch (mmol/L)	3.90 ± 0.99	4.05 ± 1.30	3.83 ± 0.79
TG (mmol/L) ^a^	1.51 (0.90–1.80)	1.74 (1.02–1.74)	1.41 (0.85–1.82)
LDL-C (mmol/L)	2.20 ± 0.71	2.32 ± 0.91	2.14 ± 0.59
HDL-C (mmol/L)	0.96 ± 0.30	0.91 ± 0.20	0.98 ± 0.34
ALB (g/L)	40.06 ± 3.56	39.14 ± 3.13	40.44 ± 3.69

*Hb* hemoglobin, *Ca* calcium, *P* phosphate, *PTH* parathyroid hormone, *SF* serum ferritin, *TIBC* Total iron binding capacity, *Tch* total cholesterol, *TG* triglycerides, *LDL-C* low-density lipoprotein cholesterol, *HDL-C* high-density lipoprotein cholesterol, *ALB* albumin

^a^Median (25th–75th percentile). *p < 0.05; **p < 0.01

### Serum klotho and cerebrovascular disease

Cox regression analysis was used to explore the independent risk factors of cerebrovascular disease in hemodialysis patients (Table 2). The results showed that serum klotho (HR(95%CI) = 0.975(0.960–0.990), *p* = 0.001) and age (HR(95%CI) = 1.104(1.038–1.174), *p* = 0.002) were the independent risk factors of cerebrovascular disease in hemodialysis patients.

**Table 2:** Cox regression analysis for incident cerebrovascular disease according to baseline variables among 88 hemodialysis patients

	Univariable		Multivariable	
	HR(95% CI)	*P*	HR(95% CI)	*P*
Age, years	1.086 (1.037–1.138)	0.001^**^	1.104 (1.038–1.174)	0.002^**^
Male, n (%)	1.667 (0.632–4.392)	0.302		
Diabetes history, n (%)	2.489 (0.927–6.680)	0.070		
Hypertension, n (%)	1.500 (0.437–5.148)	0.519		
Tobacco smoking, n (%)	1.083 (0.297–3.951)	0.904		
HD vintage (months)	1.027 (1.002–1.053)	0.033^*^	1.027 (0.996–1.058)	0.086
Klotho (pg/ml)	0.977 (0.965–0.990)	0.001^**^	0.975 (0.960–0.990)	0.001^**^
Hb (g/L)	1.006 (0.977–1.063)	0.679		
Ca (mmol/L)	1.298 (0.217–7.772)	0.775		
P (mmol/L)	0.707 (0.291–1.718)	0.444		
PTH (pg/ml)	0.999 (0.997–1.000)	0.095		
SF (ng/ml)	0.996 (0.909–1.091)	0.931		
Serum iron (umol/L)	1.000 (0.998–1.001)	0.395		
TIBC (umol/L)	0.997 (0.949–1.048)	0.913		
Tch (mmol/L)	1.249 (0.794–1.964)	0.336		
TG (mmol/L)	1.448 (0.900–2.330)	0.127		
LDL-C (mmol/L)	1.421 (0.756–2.669)	0.275		
HDL-C (mmol/L)	0.382 (0.073–2.004)	0.255		
ALB (g/L)	0.899 (0.778–1.038)	0.145		

*Hb* hemoglobin, *Ca* calcium, *P* phosphate, *PTH* parathyroid hormone, *SF* serum ferritin, *TIBC* Total iron binding capacity, *Tch* total cholesterol, *TG* triglycerides, *LDL-C* low-density lipoprotein cholesterol, *HDL-C* high-density lipoprotein cholesterol, *ALB* albumin, *HR* hazard ratio, *95% CI* 95% confidence interval

*p < 0.05; **p < 0.01

**Fig. 1:**
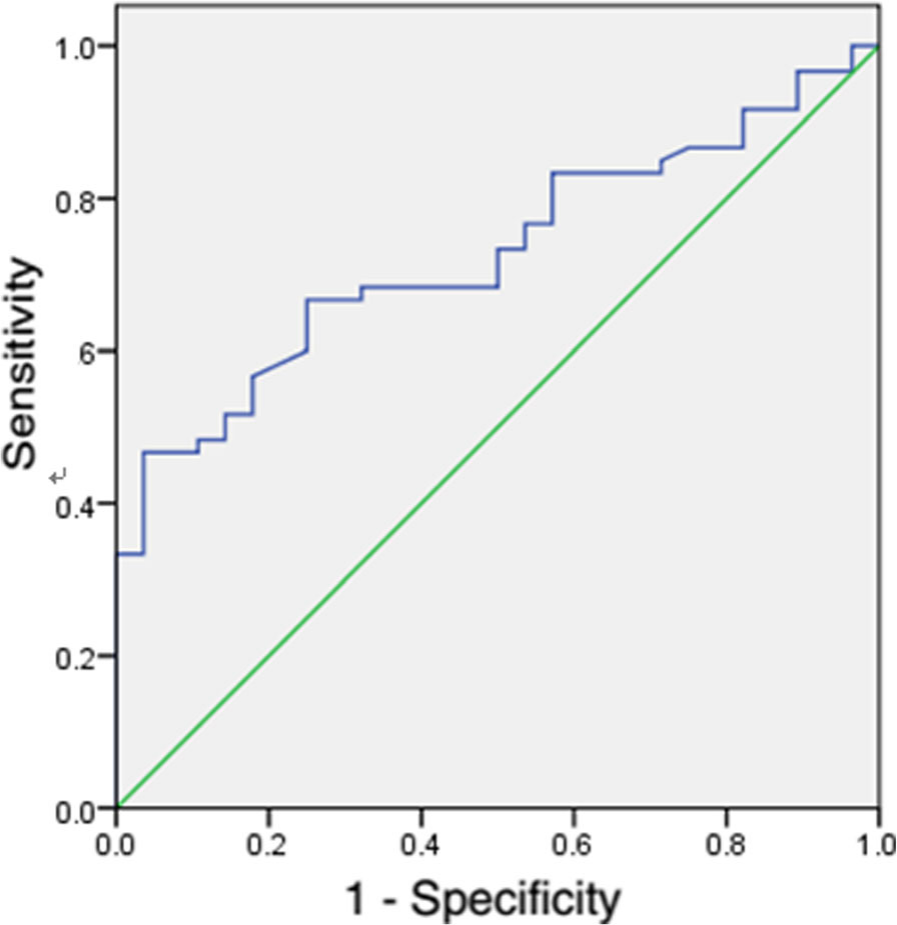
ROC curves for serum klotho predicting CVD

The ROC curve of klotho relation to cerebrovascular disease is depicted in Fig. 1. The area under the curve (AUC) for klotho and age was 0.728 (95% CI: 0.624–0.831; *P* = 0.001). The optimal cut-off value of serum klotho for predicting cerebrovascular disease was 137.22 pg/ml, with a specificity of 96.4% and a sensitivity of 46.7%.

### Correlations between the different parameters and klotho levels

The correlations of serum klotho and different parameters are shown in Table 3.Whether in the crude model or after adjusted for age, serum klotho was not significantly correlation with different parameters of the calcium-phosphate axis, iron metabolism, lipid metabolism, hemoglobin and albumin. But the level of serum klotho was negative correlation with diabetes mellitus (r = − 0.29, *p* = 0.011), and the correlation was remain unchanged after adjusted for age (r = − 2.98, *p* = 0.013).

**Table 3:** Correlations between the different parameters and klotho levels

	Crude Model		Adjusted for age	
	r	*p*	r	*p*
Ca (mmol/L)	− 0.121	0.282	0.012	0.942
P (mmol/L)	0.092	0.422	−0.134	0.272
PTH (pg/ml)	0.000	0.990	0.140	0.230
Hb (g/L)	− 0.090	0.370	−0.070	0.570
SF (ng/ml)	0.032	0.810	0.170	0.172
Serum iron (umol/L)	−0.080	0.441	−0.012	0.920
TIBC (umol/L)	−0.090	0.401	−0.082	0.491
TG (mmol/L)	−0.032	0.782	−0.112	0.351
Tch (mmol/L)	−0.041	0.713	−0.092	0.440
LDL-C (mmol/L)	0.031	0.802	−0.042	0.720
HDL-C (mmol/L)	−0.011	0.901	−0.022	0.901
ALB (g/L)	−0.092	0.461	−0.090	0.430
DM history	−0.290	0.011*	−2.980	0.013^*^
Hypertension	0.012	0.910	0.012	0.910
Tobacco smoking	0.110	0.310	0.123	0.253

*Ca* calcium, *P* phosphate, *PTH* parathyroid hormone, *Hb* hemoglobin, *SF* serum ferritin, *TIBC* Total iron binding capacity, *TG* triglycerides, *Tch* total cholesterol, *LDL-C* low-density lipoprotein cholesterol, *HDL-C* high-density lipoprotein cholesterol, *ALB* albumin, *DM* diabetes mellitus.*p < 0.05

### Serum klotho and cognitive function of patients with cerebrovascular disease

The cognitive function of patients with cerebrovascular disease was assessed with a series of tests. The performance of each cognitive test was shown in Table 4. Compared to the patients with cerebral hemorrhage, the scores of each test in patients with cerebral infarction were not significantly difference.

**Table 4:** Cognitive function of cerebrovascular disease, cerebral infarction and cerebral hemorrhage;median (25th–75th percentile)

	Cerebrovascular disease *n* = 28	Cerebral infarction *n* = 23	Cerebral hemorrhage *n* = 5
MMSE (total score)	20.05 (10.00–27.00)	17.00 (9.00–27.00)	21.00 (17.00–25.00)
HVLT-R (Immediate, words)	3.00 (1.00–5.00)	3.35 (2.00–5.00)	1.40 (0.00–3.00)
HVLT-R (Delayed, words)	2.43 (1.25–4.00)	2.48 (1.00–4.00)	2.20 (2.00–2.50)
BVMT-R (Immediate, figures)	1.75 (0.00–3.00)	1.87 (0.00–3.00)	1.20 (0.00–3.00)
VFT (animal, words)	3.79 (2.25–5.00)	4.16 (3.00–6.00)	2.40 (0.50–4.00)
Trail A (time, s)	207.35 (98.25–237.50)	186.20 (98.00–190.00)	270.8 (159.50–417.50)

*MMSE* mini-mental state examination, *HVLT-R* Hopkins Verbal Learning Test-Revised, *BVMT-R* Brief Visuospatial Memory Test-Revised, *VFT* Verbal Fluency Test

In the crude model, serum klotho was not significantly correlation with the scores of HVLT-R, BVMT-R, VFT and Trail A. But after adjusted for age, the level of serum klotho was significantly correlated with the scores of VFT (r = 0.539, *p* = 0.021) (Table 5).

**Table 5:** Correlations between the klotho levels and the scores of each cognitive test

	Crude model		Adjusted for age	
	r	*p*	r	*p*
HVLT-R (Immediate recall)	0.210	0.285	0.426	0.078
HVLT-R (Delayed recall)	0.067	0.735	0.384	0.116
BVMT-R	0.073	0.711	0.195	0.439
VFT	0.252	0.235	0.539	0.021^*^
Trail A	−0.720	0.763	−0.084	0.741

*HVLT-R* Hopkins Verbal Learning Test-Revised, *BVMT-R* Brief Visuospatial Memory Test-Revised, *VFT* Verbal Fluency Test. *p < 0.05

Seventeen cases with cerebral infarction and 5 cases with cerebral hemorrhage had cognitive impairment. Cox regression was used to assess the association between serum klotho and cognitive impairment of dialysis patients with cerebrovascular disease or cerebral infarction (Table 6). In all the patients with cerebrovascular disease or cerebral infarction, klotho was not significantly associated with cognitive impairment, neither in the crude model nor in the models adjusted for age and diabetes.

**Table 6:** Cox regression analysis the association between klotho and cognitive impairment of hemodialysis patients with cerebrovascular disease or cerebral infarction

	Cerebrovascular disease			Cerebral infarction		
	HR	95% CI	*P*	HR	95% CI	*P*
Model 1	1.003	0.988–1.017	0.732	1.006	0.991–1.022	0.417
Model 2	1.002	0.987–1.017	0.815	1.005	0.988–1.021	0.589
Model 3	1.002	0.986–1.018	0.776	1.005	0.987–1.023	0.576

Model 1, crude model; Model 2, adjusted for age; Model 3, adjusted for age and diabetes

## Discussion

The patients receiving maintain hemodialysis suffer from a serious threat of cerebrovascular disease [2]. It was reported that one-third of hemodialysis patients had experienced stroke symptoms. It means that, in addition to focus on the traditional risk factors for cerebrovascular disease, the exploration of new risk factors in dialysis patients is imminent. To our best knowledge, the current study is the first one to research the relationship between serum klotho level and cerebrovascular disease in hemodialysis patients.

The present study shows that decreased serum klotho concentration is the independently risk factor of cerebrovascular disease. Klotho, as a potential biomarker in renal disease [21], was decreased expression as the CKD progress and contribute to calcium and phosphorus metabolism. Decreased klotho protein expression possibly associated with vascular calcification and endothelial dysfunction, which might contribute to cerebrovascular disease [22–24].

Previous studies found that the suppressed expression of klotho was associated with klotho gene methylation through uremic toxins bound to DNA methyltransferase [25, 26]. Klotho gene single nucleotide polymorphism (rs650439) has also been report to be associated with differing levels of circulating klotho and the onset of stroke [27]. Genetic variants of klotho have been associated with cerebrovascular disease [28–31]. In Chinese, the relationship between genetic variants of klotho and cerebrovascular disease needs to be explored.

We found that the level of serum klotho was negatively related with diabetes mellitus after adjusted for age. The previous study also found that serum klotho level was significantly decreased in T2DM patients compared to controls [32]. Further studies confirmed that β-cell-specific expression of klotho was associated with intracellular superoxide levels, oxidative damage, apoptosis, and DNAJC3 (a marker for endoplasmic reticulum stress) [33].

Dyslipidemia was the traditional risk factor for cerebrovascular disease. In the previous studies, plasma klotho was positively correlated with HDL-C [34], and there may have a functional interrelationship between them through insulin signaling, inhibition of apoptosis, or other mechanisms [35]. Metabolic disorders of calcium and phosphorus are common in hemodialysis patients, which always lead to vascular calcification and be closely related with cerebrovascular disease. Buiten MS et al reported that serum klotho was negatively correlated with PTH and Ca in hemodialysis patients [36]. But in the present study, there was no correlation between serum klotho and HDL-C, Ca, P, or PTH in hemodialysis patients. It may because our participants are limited. Whether circulating klotho concentration is associated with lipid, calcium and phosphorus levels in hemodialysis patients needs to be further explored in a large study.

Cognitive impairment is the common sequelae of cerebrovascular disease, especially in hemodialysis patients. In klotho mutant mice at the age of 7 wk., researchers found that long- but not short-term retention of novel object recognition memory is impaired, which was the role of oxidative stress [37]. In Japanese aged 60 or over, there were statistically significant differences in cognitive function for klotho gene promoter polymorphism G-395A. Subjects with the GG type at nucleotide − 395 had lower scores of cognitive function compared to the participants with the GA/AA type [38]. In the present study, serum klotho was correlated with verbal fluency after adjusted for age, but not memory or executive function. Cox regression suggested that serum klotho is not an independent risk factor of cognitive impairment for hemodialysis patients with cerebrovascular disease .

### Strengths and limitations of the study

The present study is the first to show that serum klotho as a potential predictor of cerebrovascular disease in hemodialysis patients. But only 88 samples is our limitation. Large samples of prospective studies are needed to certify our results. Further studies are needed to explore the reason for the serum klotho decline in hemodialysis patients. Prospective studies will be needed to determine whether treatment of klotho deficiency may be a promising strategy to decrease the burden of comorbidity in hemodialysis patients.

## Conclusions

The serum klotho level is a potential predictor of cerebrovascular disease in hemodialysis patients, but it is not an independent risk factor of cognitive impairment for hemodialysis patients with cerebrovascular disease.

## Abbreviations

95% CI: 95% confidence interval
ALB: Albumin
BVMT-R: Brief Visuospatial Memory Test-Revised
Ca: Calcium
CKD: Chronic kidney disease
CVD: cardiovascular disease
DM: Diabetes mellitus
ESRD: End-stage renal disease
Hb: Hemoglobin
HDL-C: High-density lipoprotein cholesterol
HR: hazard ratio
HVLT-R: Hopkins Verbal Learning Test-Revised
LDL-C: Low-density lipoprotein cholesterol
MMSE: Mini-mental state examination
P: Phosphate
PTH: Parathyroid hormone
SBI: silent brain infarction
SF: Serum ferritin
Tch: Total cholesterol
TG: Triglycerides
TIBC: Total iron binding capacity
VFT: Verbal Fluency Test
